# Relationship between post-cardiac arrest myocardial oxidative stress and myocardial dysfunction in the rat

**DOI:** 10.1186/s12929-014-0070-6

**Published:** 2014-08-19

**Authors:** Fernanda Schäfer Hackenhaar, Francesca Fumagalli, Giovanni Li Volti, Valeria Sorrenti, Ilaria Russo, Lidia Staszewsky, Serge Masson, Roberto Latini, Giuseppe Ristagno

**Affiliations:** 1 Department of Biophysics, Universidade Federal of Rio Grande do Sul, Porto Alegre, Brazil; 2 IRCCS - Istituto di Ricerche Farmacologiche “Mario Negri”, Milan, Italy; 3 Department of Clinical and Molecular Biomedicine, University of Catania, Catania, Italy; 4 Euromediterranean Institute of Science and Technology, Palermo, Italy; 5 Department of Drug Sciences, University of Catania, Catania, Italy; 6 Department of Cardiovascular Research, IRCCS - Istituto di Ricerche Farmacologiche “Mario Negri”, Via La Masa 19 - 20156, Milan, Italy

**Keywords:** Cardiopulmonary resuscitation, Myocardial injury, Oxidative damage, Isoprostanes, 8-hydroxyguanosine

## Abstract

**Background:**

Reperfusion after resuscitation from cardiac arrest (CA) is an event that increases reactive oxygen species production leading to oxidative stress. More specifically, myocardial oxidative stress may play a role in the severity of post-CA myocardial dysfunction. This study investigated the relationship between myocardial oxidative stress and post-CA myocardial injury and dysfunction in a rat model of CA and cardiopulmonary resuscitation (CPR). Ventricular fibrillation was induced in 26 rats and was untreated for 6 min. CPR, including mechanical chest compression, ventilation, and epinephrine, was then initiated and continued for additional 6 min prior to defibrillations. Resuscitated animals were sacrificed at two h (n = 9), 4 h (n = 6) and 72 h (n = 8) following resuscitation, and plasma collected for assessment of: high sensitivity cardiac troponin T (hs-cTnT), as marker of myocardial injury; isoprostanes (IsoP), as marker of lipid peroxidation; and 8-hydroxyguanosine (8-OHG), as marker of DNA oxidative damage. Hearts were also harvested for measurement of tissue IsoP and 8-OHG. Myocardial function was assessed by echocardiography at the corresponding time points. Additional 8 rats were not subjected to CA and served as baseline controls.

**Results:**

Compared to baseline, left ventricular ejection fraction (LVEF) was reduced at 2 and 4 h following resuscitation (p < 0.01), while it was similar at 72 h. Inversely, plasma hs-cTnT increased, compared to baseline, at 2 and 4 h post-CA (p < 0.01), and then recovered at 72 h. Similarly, plasma and myocardial tissue IsoP and 8-OHG levels increased at 2 and 4 h post-resuscitation (p < 0.01 vs. baseline), while returned to baseline 72 h later. Myocardial IsoP were directly related to hs-cTnT levels (r = 0.760, p < 0.01) and inversely related to LVEF (r = -0.770, p < 0.01). Myocardial 8-OHG were also directly related to hs-cTnT levels (r = 0.409, p < 0.05) and inversely related to LVEF (r = -0.548, p < 0.01).

**Conclusions:**

The present study provides evidence that lipid peroxidation and DNA oxidative damage in myocardial tissue are closely related to myocardial injury and LV dysfunction during the initial hours following CA.

## Background

The prognosis of cardiac arrest after cardiopulmonary resuscitation (CPR) remains poor, with more than 70% of resuscitated patients dying within 72 h after return of spontaneous circulation (ROSC), due to the well described post-cardiac arrest syndrome [1]. Severe heart contractile failure due to post-resuscitation myocardial dysfunction has been implicated as the most important mechanism accounting for early death after ROSC [2].

Due to the complexity and interplay of events occurring during cardiac arrest and after ROSC, mechanisms involved in post-resuscitation myocardial dysfunction are not completely understood, and new injury pathways have been described [3]. Cardiac arrest is a global ischemic event followed by a whole body reperfusion when ROSC is achieved. Indeed, ischemia-reperfusion (I/R) is a process characterized by reactive oxygen species (ROS) generation, which starts during ischemia and is further exacerbated by defibrillation attempts and subsequently by return of oxygenated blood to the tissues after ROSC [4]–[6]. This increase in ROS production leads to oxidation of cell macromolecules, such as lipids, proteins, and possibly DNA, in a process overall named oxidative stress [7],[8]. The oxidative stress events are recognized to participate in processes leading to acute inflammatory response, cell damage, mitochondrial dysfunction, decrease in nitric oxide (NO) availability, and ultimately cell apoptosis and death [4]–[7],[9]–[12]. In the heart, these events may impair the normal function of cardiomyocytes, contributing to the severity of post-cardiac arrest myocardial dysfunction [4].

There are no published studies comprehensively assessing the relations over time between different markers of oxidative stress in plasma and myocardium, cardiac troponins (cTn) and left ventricular (LV) function. The aim of this study was to investigate the severity of myocardial oxidative stress following resuscitation in a rat model of cardiac arrest and CPR and its relationship with the severity of post-cardiac arrest myocardial injury and dysfunction. We hypothesized that myocardial oxidative injury may contribute to the severity of post-cardiac arrest myocardial injury and dysfunction.

## Methods

Procedures involving animals and their care were in compliance with national (D.L. n. 116, G.U., suppl. 40, 18 February 1992, Circolare no. 8, G.U., 14 Luglio 1994) and international laws and policies (EEC Council Directive 86/609, OJL 358, 1, December 12, 1987; Guide for the Care and Use of Laboratory Animals, US National Research Council, 1996). Approvals of the studies were obtained by the local institutional review board committee and governmental institution.

### Animal preparation

Thirty-four male Sprague-Dawley rats weighing 470 ± 26 g were fasted overnight except for free access to water. The details of the animal preparation were published previously [3]. In brief, the animals were anesthetized by intraperitoneal injection of pentobarbital (50 mg/kg), and additional doses (10 mg/kg) were administrated at intervals of approximately 1 h or when required to maintain anesthesia. The trachea was orally intubated with a 14-gauge cannula. A PE-50 catheter (Becton Dickinson, FranklinLakes, NJ) was advanced into the descending aorta from the left femoral artery for measurement of arterial pressure and sampling arterial blood. Through the left external jugular vein, another PE-50 catheter was advanced into the right atrium for measurement of right atrial pressures. Aortic and right atrial pressures were measured with reference to the mid chest with high-sensitivity transducers. A 3-F PE catheter (model C-PMS-301 J, Cook Critical Care, Bloomington, IN) was advanced through the right external jugular vein into the right atrium. A pre-curved guide wire supplied with the catheter was then advanced through the catheter into the right ventricle and confirmed by endocardial electrocardiogram for inducing ventricular fibrillation (VF). All of the catheters were flushed intermittently with saline containing 2.5 IU/mL of bovine heparin. A conventional lead II electrocardiogram (ECG) was continuously monitored. Temperature was continuously monitored with the aid of a rectal probe and maintained at 37 ± 0.5°C throughout the experiment.

### Experimental procedures

Fifteen minutes prior to inducing VF, baseline measurements were obtained and mechanical ventilation was initiated with an inspired FiO_2_ of 0.21. VF was electrically induced with progressive increases in 60-Hz current to a maximum of 4 mA delivered to the right ventricular endocardium. The current flow was continued for 3 min to prevent spontaneous defibrillation. Mechanical ventilation was stopped after the onset of VF. Precordial compression was begun after 6 min of untreated VF with a pneumatically driven mechanical chest compressor as previously described [3]. Coincident with the start of precordial compression, animals were mechanically ventilated at a frequency of 50/min with a tidal volume 0.6 ml/100 g and a FiO_2_ of 1.0. Precordial compression was maintained at a rate of 200/min with equal compression-relaxation duration (i.e., 50% duty cycle) and a depth of compression equal to 25% of the animal’s antero-posterior chest diameter. Epinephrine (0.02 mg/kg) was injected into the right atrium 2 min after the start of precordial compression. After 6 min of CPR, resuscitation was attempted with up to three 2 Joule defibrillations (CodeMaster XL, Philips Heartstream, Seattle, WA). Successful resuscitation was defined as the return of supraventricular rhythm with a mean aortic pressure (MAP) > 50 mm Hg for a minimum of 5 min. Following resuscitation, animals were monitored for 4 h. All the catheters and the endotracheal tubes were then removed. The animals were returned to their cages and were observed for up to 3 days after resuscitation. Animals were sacrificed with an intraperitoneal injection of pentobarbital sodium (150 mg/kg) at different intervals: baseline, before cardiac arrest; 2 h post-resuscitation; 4 h post-resuscitation; and 72 h post-resuscitation. Plasma was withdrawn for biochemical analyses. Blood was collected into EDTA-tubes and centrifuged for 15 min at 3000 rpm at 4°C. Plasma samples were then stored at -80°C. The heart was quickly removed from the thoracic cavity, blotted and frozen at -80°C for further biochemistry.

### Measurements

Aortic and right atrial pressures, and ECG were continuously recorded on a personal computer-based data acquisition system supported by CODAS hardware and software (DataQ, Akron, OH). Coronary perfusion pressure (CPP) was calculated as the difference between aortic and time-coincident right atrial pressures [3]. Transthoracic echocardiography was performed at 3 and 72 h after resuscitation using SSD-5500 echo machine (Aloka, Tokyo, Japan) equipped with a 13 MHz linear array transducer at high frame rate imaging (102 Hz) and a 7.5 MHz phased array probe for pulsed-wave, color and tissue Doppler imaging. Echocardiographic images were obtained from parasternal short and long-axis views and from apical views. End-diastolic and end-systolic wall thicknesses, systolic wall thickening, LV internal dimensions and fractional shortening were measured and calculated. LV volumes and ejection fraction (EF) were calculated by the modified single plane Simpson’s rule from the parasternal long-axis view as previously reported [3]. Aortic outflow velocities were measured from a 5 apical chamber view by pulsed-wave Doppler and LV cardiac output (CO) and stroke volume (SV) were calculated [3].

Plasma high sensitivity cTnT (hs-cTnT) levels were assessed with an electrochemiluminescence assay (ECLIA, Elecsys 2010 analyzer, Roche Diagnostics, Germany). Isoprostanes (IsoP) and 8-hydroxyguanosine (8-OHG) were assessed by commercially available ELISA Kits (Cayman Chemical, Ann Arbor, MI, USA) according to the manufacturer’s instruction. Plasma glutathione *S*-transferase (GSH) was measured by a spectrophotometric assay, in accordance with the method of Hu, as previously reported [13]. Briefly, the method was based on the reaction of thiol groups with 2,2-dithio-bis-nitrobenzoic acid (DTNB) in absolute ethanol to give a colored compound absorbing at λ = 412 nm. Removal of proteins was performed with an excess of absolute ethanol, followed by centrifugation at 3000 g for 10 min at room temperature. Asymmetric dimethylarginine (ADMA) concentration was also measured by a commercially available ELISA Kit (DLD Diagnostika, Hamburg, Germany). For assessment of dimethylarginine dimethylaminohydrolase (DDAH), tissues were homogenized in 0.1 M phosphate buffer, pH 6.5, containing 2 mM mercapto-ethanol and protease inhibitor cocktail (1:1000). Homogenates were centrifuged at 5000 g for 60 min, and supernatants were collected for DDAH enzyme activity assay. DDAH enzyme activity was assayed by determining L-citrulline formation in a 96-well microtiter plate. One unit of enzyme activity was defined as the amount of enzyme catalyzing the formation of 1 mol L-citrulline/min at 37°C.

### Statistical analysis

One sample Kolmogorov–Smirnov Z test was used to confirm normal distribution of the data. Comparisons among time-based measurements within groups were performed by one-way ANOVA with Fisher’s LSD post-hoc test. Linear correlations between parametric variables were calculated using the Pearson correlation coefficient. Spearman test was performed for the non-parametric variable correlation analyses. Data are reported as mean ± SEM, except for hs-cTnT, presented as median [25-75 percentile]. A 2-tail p < 0.05 was considered as statistically significant. The analyses were performed using GraphPad Prism 6.

## Results

A total of 34 rats were included in the study. Eight control rats were not subjected to cardiac arrest and served as baseline, while the other 26 underwent cardiac arrest and CPR. Among the 26 cardiac arrest rats, 23 were successfully resuscitated (88%) and were assigned to be sacrificed at 2 h (n = 8), 4 h (n = 9), and 72 h (n = 8) after resuscitation. No differences in body weight and number of defibrillations and duration of CPR prior to ROSC were observed among the groups (Table 1). Among the 8 rats undergoing to 72 h of post-resuscitation observation, 5 (63%) survived till the end of the observational period.

**Table 1:** Resuscitation outcome

	**Baseline**	**PR 2 h**	**PR 4 h**	**PR 72 h**
	**(n = 8)**	**(n = 9)**	**(n = 6)**	**(n = 8)**
**Body Weight, g**	475 ± 13	477 ± 7	472 ± 7	458 ± 6
**Defibrillations to ROSC, n**	—	4 ± 1	3 ± 1	2 ± 1
**Time to ROSC, sec**	—	396 ± 16	373 ± 10	360 ± 1

PR, post resuscitation; ROSC, return of spontaneous circulation.

Data shown as mean ± SEM.

### Hemodynamics and myocardial function

**Figure 1:**
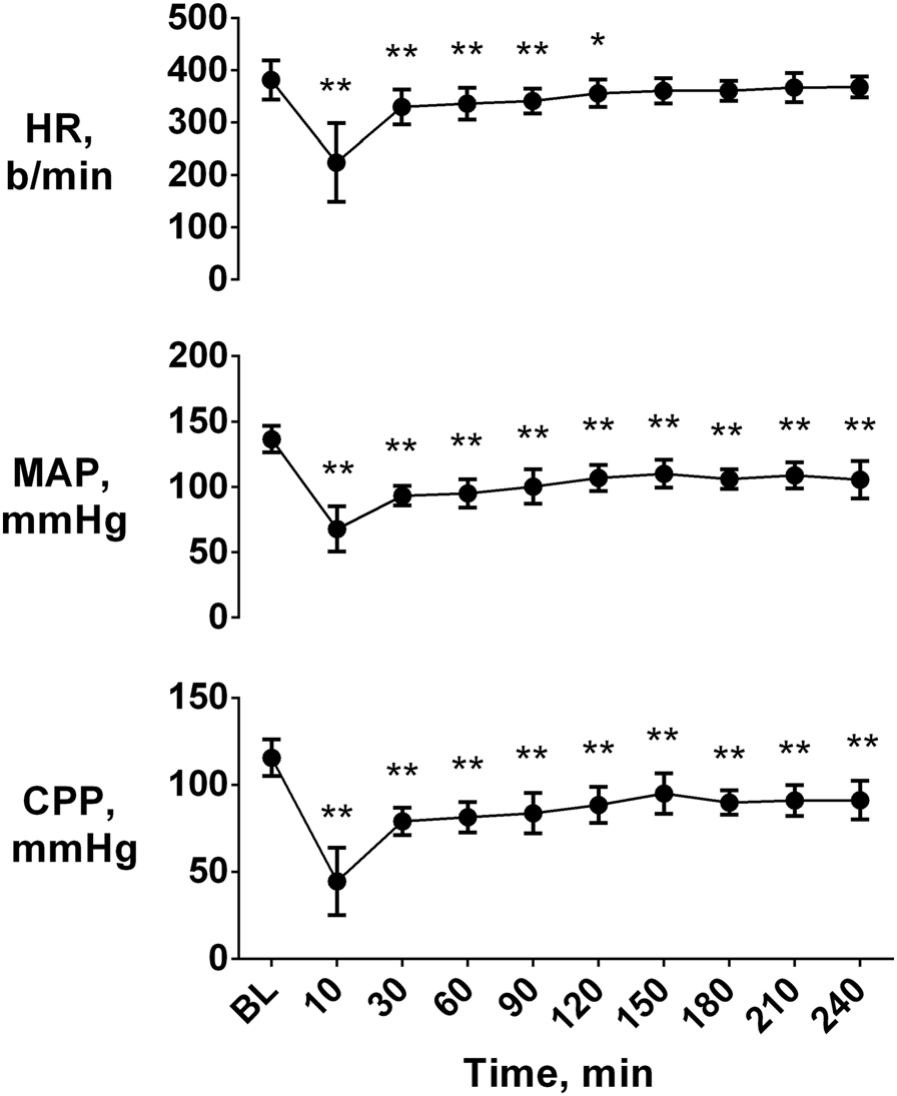
**Hemodynamics.** Heart rate (HR), mean arterial pressure (MAP) and coronary perfusion pressure (CPP) at baseline (BL) and post-resuscitation. Data are shown as mean ± SEM; *p < 0.05 and **p < 0.01 vs. BL.

**Figure 2:**
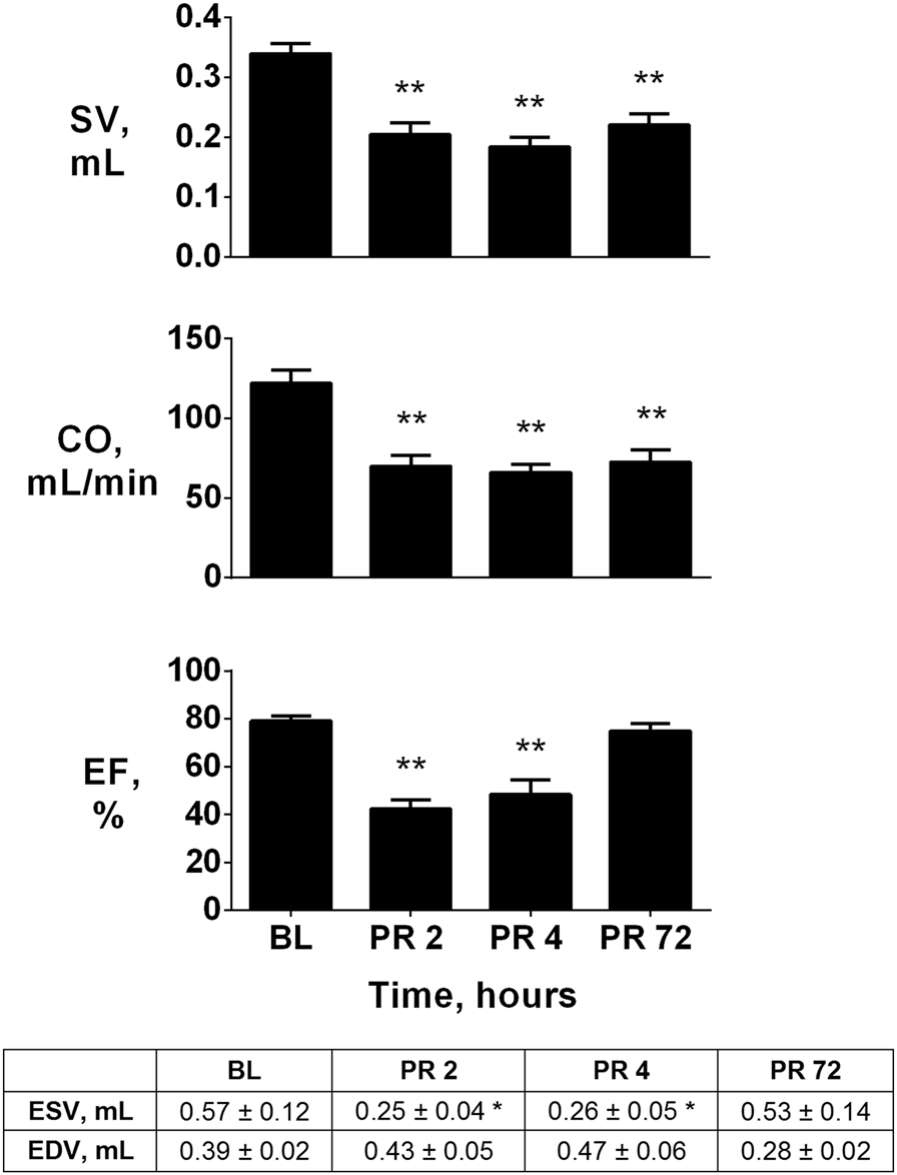
**Myocardial function.** Left ventricle stroke volume (SV), cardiac output (CO) and ejection fraction (EF) at baseline (BL) and post-resuscitation (PR). The bottom table reports data on left ventricle end systolic volume (ESV) and end diastolic volume (EDV). Data are shown as mean ± SEM; *p < 0.05 and **p < 0.01 vs. BL.

Each rat developed a marked post-resuscitation myocardial dysfunction during the 4 h of observation, which regressed 72 h later. Indeed, heart rate was significantly reduced during the first 2 h post-resuscitation compared to baseline (Figure 1), while MAP and CPP remained decreased for the entire 4 h post-resuscitation (p < 0.01 vs. baseline, Figure 1). Similarly to hemodynamics, LV end systolic volume and EF were also significantly decreased at 2 and 4 h post-resuscitation (p < 0.01 vs. baseline), while returned to baseline 72 h later (Figure 2). Resuscitated rats also presented markedly reduced LV SV and CO (p < 0.01 vs. baseline, Figure 2) at 2, 4, and 72 h post-resuscitation.

Changes observed in post-resuscitation myocardial function, expressed as LVEF, were paralleled by the concurrent changes in circulating levels of hs-cTnT (Table 2). Plasma levels of hs-cTnT, in fact, significantly increased at 2 and 4 h post-resuscitation (p < 0.01 vs. baseline), and then normalized at 72 h (Table 2). Hs-cTnT levels inversely correlated with LVEF (r = -0.89, p < 0.01).

**Table 2:** Circulating oxidative stress biomarkers and asymmetrical dimethylarginine (ADMA) and dimethylarginine dimethylaminohydrolase (DDAH)

	**Baseline**	**PR 2 h**	**PR 4 h**	**PR 72 h**
	**(n = 8)**	**(n = 9)**	**(n = 6)**	**(n = 8)**
**Plasma hs-cTnT, ng/L**	61 [20-91]	3732 [3277-6218]**	3469 [2428-4930]*	44 [39-1610]
**Plasma IsoP, pg/mL**	235.6 ± 28.8	206.2 ± 14.9	306.6 ± 21.6*	216.6 ± 30.0
**Plasma 8-OHG, pg/mL**	742.9 ± 79.3	1044.5 ± 71.1*	914.9 ± 104.1	846.9 ± 14.7
**Plasma GSH, μmol/mL**	0.146 ± 0.030	0.099 ± 0.004	0.095 ± 0.013	0.136 ± 0.023
**Plasma ADMA, μmol/L**	0.767 ± 0.10	0.641 ± 0.07	0.468 ± 0.06*	0.601 ± 0.07
**Myocardial ADMA, nmol/mg prot**	0.083 ± 0.01	0.052 ± 0.01	0.043 ± 0.01	0.080 ± 0.02
**Myocardial DDAH, nmol/mg prot**	77.5 ± 12.7	73.5 ± 7.2	79.0 ± 10.4	90.0 ± 12.8

PR, post resuscitation; 8-OHG, 8-hydroxyguanosine; hs-cTnT, high-sensitivity cardiac troponin T; IsoP, isoprostanes; GSH, glutathione *S*-transferase.

Parametric data are shown as mean ± SEM; Non-parametric data are shown as median [25-75 percentile].

*p < 0.05 vs. Baseline; **p < 0.01 vs. Baseline.

### Oxidative stress biomarkers

**Figure 3:**
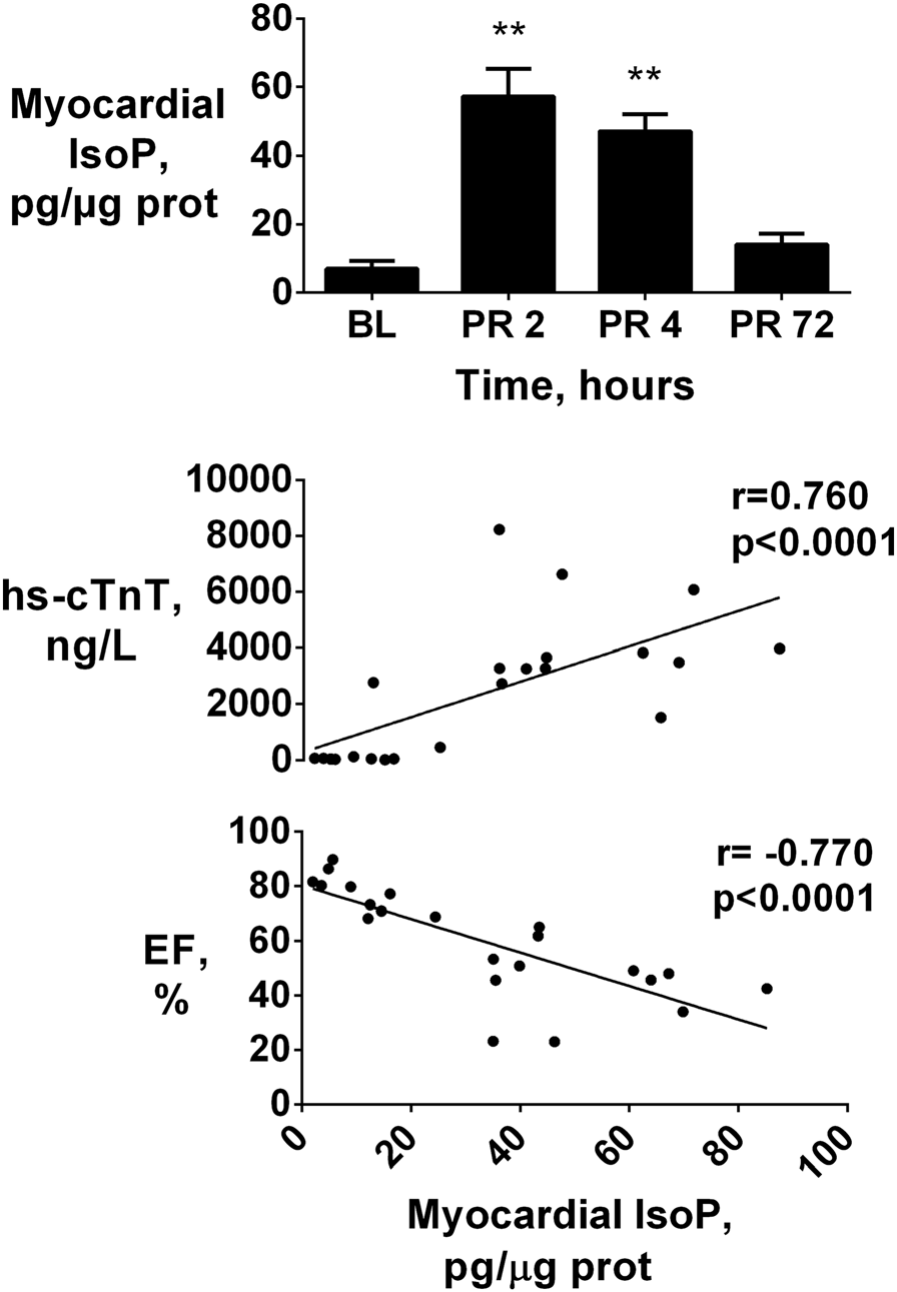
**Myocardial isoprostanes vs. myocardial injury and function.***Upper:* Myocardial isoprostanes (IsoP) levels at baseline (BL) and post resuscitation (PR), reported as mean ± SEM, **p < 0.01 vs. BL. *Bottom:* relation between IsoP and high-sensitivity cardiac troponin (hs-cTnT) and left ventricle ejection fraction (LVEF).

Plasma levels of IsoP, a marker of lipid peroxidation, significantly increased at 4 h post-resuscitation and recovered 72 h later (p < 0.05 vs. baseline, Table 2). Changes in myocardial tissue IsoP were more evident compared to those in plasma (Figure 3). Myocardial IsoP markedly increased at 2 and 4 h post-resuscitation (p < 0.01 vs. baseline, Figure 3), while levels returned to baseline values 72 h later. Myocardial IsoP were directly related to plasma hs-cTnT (r = 0.760, p < 0.0001, Figure 3) and inversely related to LV EF (r = -0.770, p < 0.0001, Figure 3).

**Figure 4:**
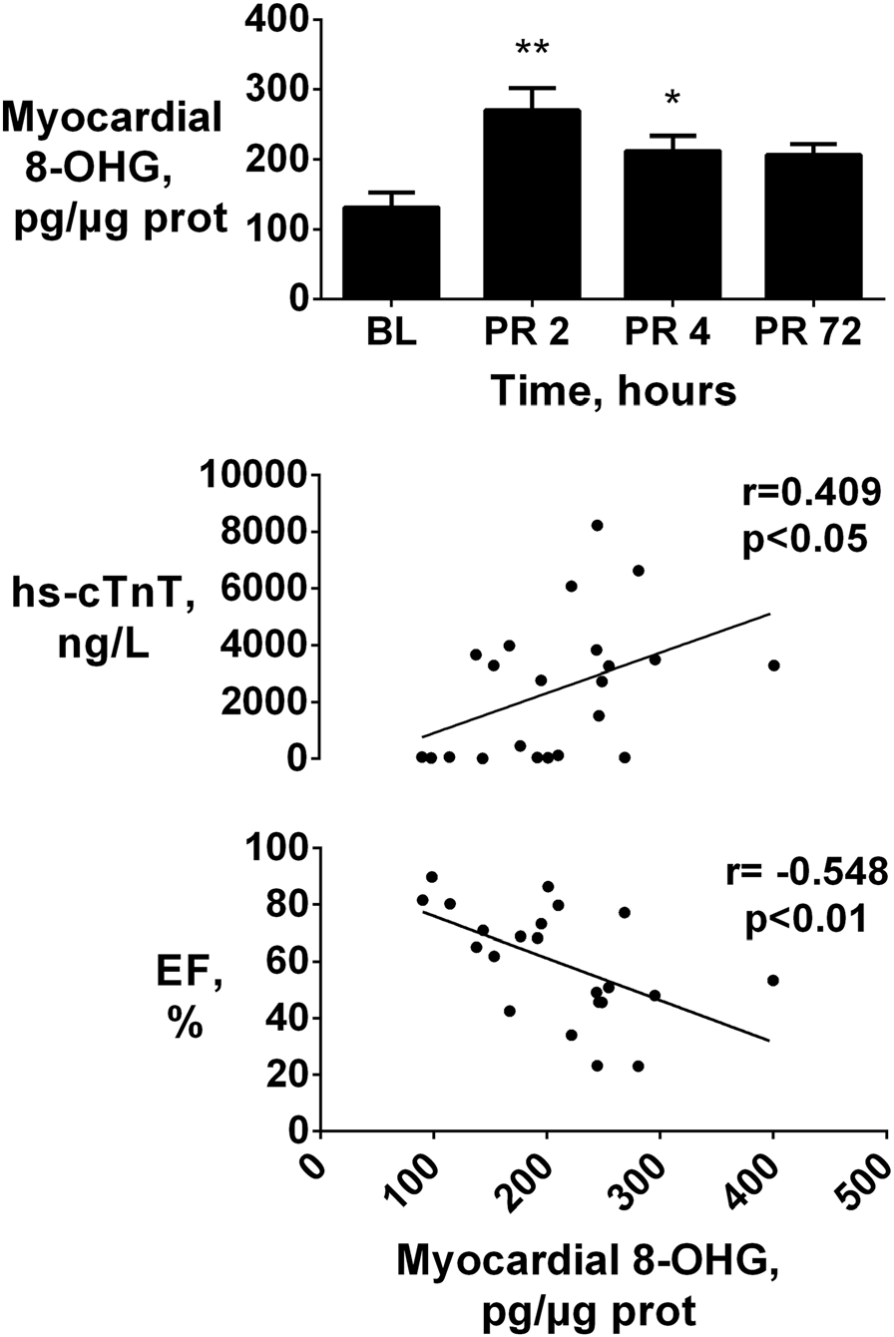
**Myocardial 8-hydroxyguanosine vs. myocardial injury and function.***Upper:* Myocardial 8-hydroxyguanosine (8-OHG) levels at baseline (BL) and post resuscitation (PR), reported as mean ± SEM, **p < 0.01 and *p < 0.05 vs. BL. *Bottom:* relation between 8-OHG and high-sensitivity cardiac troponin (hs-cTnT) and left ventricle ejection fraction (EF).

Plasma levels of 8-OHG, a marker of DNA damage, significantly increased at 2 h post-resuscitation and recovered within the following 2 h of observation (p < 0.05 vs. baseline, Table 2). Changes in myocardial tissue 8-OHG were more evident compared to those in plasma (Figure 4). Myocardial 8-OHG markedly increased at 2 and 4 h post-resuscitation (p < 0.01 vs. baseline, Figure 4), while levels returned to baseline values 72 h later. Myocardial 8-OHG were directly related to plasma hs-cTnT (r = 0.409, p < 0.05, Figure 4) and inversely related to LVEF (r = -0.548, p < 0.01, Figure 4).

Plasma levels of GSH slightly decreased at 2 and 4 h post-resuscitation (p not significant) and recovered 72 h later (Table 2).

ADMA, an endogenous inhibitor of nitric oxide synthase (NOS), was assayed in plasma and myocardial tissue. Plasma levels of ADMA were significantly decreased at 4 h post-resuscitation compared to baseline (p < 0.05, Table 2). Similarly, there was a trend towards decreases in myocardial ADMA levels during the 4 h of post-resuscitation (p = 0.05 vs. baseline, Table 2), while it recovered to baseline values 72 h later.

Decreases in myocardial ADMA were paralleled by concurrent changes in myocardial DDAH, the enzyme that degrades ADMA (Table 2). However, only a trend towards increases in myocardial DDAH was observed at 4 and 72 h post-resuscitation (p not significant, Table 2).

## Discussion

The present study describes the entity of circulating and myocardial tissue levels of oxidative stress markers during the initial hours post-resuscitation and it suggests a contribution of oxidative damage to the severity of post-cardiac arrest myocardial injury and dysfunction. Indeed, IsoP and 8-OHG levels in the myocardial tissue significantly increased at 2 and 4 h post-resuscitation, and these increases related to the increased hs-cTnT levels, a specific marker of myocardial injury, and to the impaired LVEF, a marker of myocardial dysfunction. Plasma levels of circulating IsoP and 8-OHG were also significantly increased after resuscitation, indicating a concurrent condition of whole body oxidative stress.

Post-cardiac arrest myocardial dysfunction contributes to the early deaths after resuscitation from cardiac arrest [2]. It is characterized by a compromised LV systolic function with low CO and arterial pressure. In patients who survive, this myocardial dysfunction is transient and fully recovers during the following 2-3 days after ROSC [2]. cTn are regulatory proteins belonging to the heart’s contractile apparatus that regulates the calcium-mediated interaction of actin and myosin. Accordingly, cTn release is commonly observed after resuscitation from cardiac arrest [14]. The release of cTn from the myocardium might reflect an ongoing myocyte cell death as well as other events, i.e. myocyte stretching with transient loss of cell membrane integrity, and is related to the severity of myocardial dysfunction [14],[15]. In our study, we observed significant reductions in post-cardiac arrest LV EF, at 2 and 4 h following ROSC, with a subsequent recovery 72 h later. A strong relationship between LV EF and circulating hs-cTnT was confirmed at different time points following ROSC. Normal LV function at 72 h post ROSC indicates that myocardial viability was preserved even in the presence of previous hs-cTnT degradation. This suggests that post-resuscitation myocardial dysfunction is largely reversible in the present model.

ROS formation, including free radical superoxide, hydrogen peroxide, and hydroxyl radical, occurs in low levels under physiological conditions, but it is exacerbated as a consequence of I/R injury [5]–[8]. Under this condition of I/R, ROS are produced in myocardial and other tissues by several mechanisms, involving actions of xanthine oxidase, NOS, and nicotinamide adenine dinucleotide phosphate oxidase [16],[17],[3]. Consequently, this exacerbated ROS production creates an imbalance between oxidant and antioxidant mechanisms inside the cells, which may contribute to post-cardiac arrest myocardial dysfunction [18]. Inflammatory cell infiltration in myocardial tissue is another well known process after I/R that contributes to ROS generation, leading to apoptosis of cardiomyocytes [4]. Finally, mitochondria play also a crucial role in increasing ROS generation after I/R, due to an uncoupling of electron transport chain after reperfusion [19]. The consequent ROS increase in the mitochondria, in addition to the reperfusion-related calcium overload, further activates the apoptotic cell death pathways [20],[21]. Indeed, earlier studies have reported that mitigating ROS production, by limiting myocardial injury, may represent a future target for new therapeutic interventions [4]. Indeed, a recent experimental study confirmed that administration of an antioxidant after ROSC, i.e. ascorbic acid, was able to reduce lipid peroxidation, resulting in an improved post-resuscitation myocardial dysfunction and ultimately in a greater survival [12].

In our study we assessed oxidative stress after CPR by measuring markers of lipid oxidation, namely IsoP, and a marker of DNA oxidation, namely 8-OHG. IsoP are stable products of arachidonic acid oxidation and are released from cells undergoing oxidative stress [22]. Indeed, two- to three-fold increases in IsoP have been previously reported in plasma after resuscitation from cardiac arrest in pigs, and in cardiac tissue after myocardial I/R in mice [6],[23]. IsoP present important biological activity [7]. The oxidation of these lipids, in fact, impairs cell membrane stability, leading to altered permeability, endothelial dysfunction, and consequent cell damage [5],[24]. In a model of myocardial I/R in rats, pretreatment with F-isoprostane induced a five-fold increased endothelin-1 release, impaired LV developed pressure, and increased infarct size compared to control animals [24]. Indeed, in our experiment, both plasma and myocardial tissue IsoP significantly increased after resuscitation. Nevertheless, myocardial IsoP increase was more evident compared to plasma increase and highly correlated with hs-cTnT releases and LV dysfunction over time.

8-OHG is a specific biomarker of oxidative damage to DNA [25],[26]. Similarly to our results, increases in urinary 8-OHG have been reported in patients with acute myocardial infarction, with peak levels at 4 h after reperfusion therapy and a return to baseline levels within 24 h [27]. Whether oxidative stress-related DNA damage and altered gene expression may contribute to LV dysfunction and remodeling after an ischemic event is not clear [28]. A report in a rat model of I/R demonstrated increased 8-OHG in nuclei of cardiomyocytes after reperfusion, which, however, seemed not to have any relation with the altered LV function [9]. Other studies, instead, reported a correlation between the oxidative damage to mitochondrial DNA and the severity of LV dilatation and reduced contractility in mice subjected to myocardial infarction [28]. Similarly to our results, high serum and urinary levels of 8-OHG were observed in patients with heart failure and further increases in 8-OHG occurred for higher New York Heart Association (NYHA) functional class, such that 8-OHG was suggested as prognostic tool for risk stratification in these patients [25],[26]. To the best of our knowledge, this is the first study relating 8-OHG to post-cardiac arrest LV dysfunction. Our results suggest a relationship between 8-OHG and myocardial injury and function, similarly to IsoP. Increase in post-resuscitation 8-OHG levels were, however, of lesser extent compared to those in IsoP.

Oxidative stress following cardiac arrest is a systemic event, such that oxidative stress biomarkers are generated in all tissues, as products of the oxidation of molecules present in every cell types [27],[29]. Such a condition of whole body post-resuscitation oxidative stress is indeed supported by the significant increases in plasma IsoP and 8-OHG together with the concurrent reduction in plasma GSH, a known endogenous inactivator of oxygen-derived highly reactive species [13]. Consequently, in contrast to plasma levels, dosage of oxidative stress biomarkers directly in myocardial tissue was a more reliable marker for myocardial damage, and better represented the time-dependent oxidative stress in our study. Myocardial IsoP and 8-OHG increases were directly related with hs-cTnT and inversely related with LVEF. Whether the observed relation between tissue IsoP and 8-OHG and established markers of cardiac injury such as cTn and LVEF, is causal needs to be further investigated.

Accordingly, our findings indicate a possible contribution of oxidative stress to post-cardiac arrest myocardial injury and dysfunction. After I/R, endothelial dysfunction and inhibition of NOS with reduced NO availability are commonly observed, due to oxidative stress [11]. NO is an endothelium-derived vasoactive factor produced by NOS, that plays important roles in modulating coronary vascular tone and tissue perfusion [30]. NO, however, is also known to be able to react with free radical superoxide producing peroxynitrite, a more powerful oxidating reactive specie, which worsens tissue damage by protein nitration [31]. ADMA is an endogenous inhibitor of NOS activity, present in high concentration inside the endothelial cells [32],[33]. In a recent study in coronary artery bypass grafting patients, cardioplegic arrest was associated with decreased plasma levels of ADMA; the mechanisms involved in this process remained, however, unexplained [34]. Similarly, in our study, decreases in both plasma and myocardial tissue levels of ADMA after cardiac arrest were observed and paralleled reductions in LVEF and increases in hs-cTnT. Decreases in myocardial ADMA were paralleled and explained by concurrent increases in its degrading enzyme DDAH [33]. It can be hypothesized that these ADMA decreases may potentially exacerbate post-cardiac arrest myocardial dysfunction. Decreases in ADMA may, in fact, lead to decrease in NOS inhibition and consequent greater NO production and nitrosative stress [35]. Moreover, besides inhibiting NO synthesis, ADMA can directly induce oxidative stress and cell apoptosis, and participate in inflammatory reactions [33],[34],[36],[37]. Indeed, by inhibiting endothelial NOS, ADMA might increase the risk of vascular inflammation and thrombosis, such that elevated plasma ADMA has been reported to be a risk factor for cardiovascular morbidity and mortality in patients with cardiovascular disease [33]. Thus, we cannot exclude that decrease in ADMA may, instead, reflect an adaptive change aimed to protect from oxidative stress.

Some limitations need to be taken into account when interpreting our results. First, the study was performed in healthy animals with no previous myocardial damage. Second, this study is a descriptive report and no direct mechanisms assessing the connection between oxidative stress and myocardial damage and dysfunction were shown, nor effects from inhibition of oxidative stress pathways on post-resuscitation myocardial dysfunction. Accordingly, further interventional experimental studies are underway. Nevertheless, the results demonstrated the clear relationship between oxidative stress and post-resuscitation hs-cTnT release and LV dysfunction. Moreover, both circulating and tissue levels of oxidative stress markers have been reported overtime post-resuscitation, providing new insights in the time-course of these biomarkers.

## Conclusions

The present study in a rat model of CPR demonstrated the possible contribution of oxidative damage to post-cardiac arrest syndrome. It provided evidence that lipid peroxidation and DNA oxidative damage in myocardial tissue are closely related to myocardial injury, represented by hs-cTnT release, and LV dysfunction, represented by reduced EF, during the initial hours following resuscitation from cardiac arrest.

## Abbreviations

8-OHG: 8-hydroxyguanosine

ADMA: Asymmetric dimethylarginine

CO: Cardiac output

CPP: Coronary perfusion pressure

CPR: Cardiopulmonary resuscitation

cTn: Cardiac troponins

DDAH: Dimethylarginine dimethylaminohydrolase

ECG: Electrocardiogram

EF: Ejection fraction

GSH: Glutathione *S*-transferase

hs-cTnT: High sensitivity cardiac troponin

I/R: Ischemia/reperfusion

IsoP: Isoprostanes

LV: Left ventricle

MAP: Mean arterial pressure

NO: Nitric oxide

ROS: Reactive oxygen species

ROSC: Return of spontaneous circulation

SV: Stroke volume

VF: Ventricular fibrillation

## Competing interests

The authors declare that they have no competing interests.

## Authors’ contributions

FSH participated in the study design, performed the statistical analysis, interpreted the results, and drafted the manuscript; FF participated in the study design and carried out all the *in vivo* studies; GLV participated in the study design and in the oxidative stress biomarker measurements, and helped to draft the manuscript; VS carried out the oxidative stress biomarker measurements; IR carried out the echocardiographic exams; LS participated in echocardiographic exams and helped to draft the manuscript; SM participated in the plasma cardiac troponins measurements, in the interpretation of the results, and helped to draft the manuscript; RL participated in the study design and helped to draft the manuscript; GR conceived the study and participated in the statistical analysis, interpretation of the results, and drafting of the manuscript. All authors read and approved the final manuscript.
